# Predicting ADHD by Assessment of Rutter’s Indicators of Adversity in Infancy

**DOI:** 10.1371/journal.pone.0157352

**Published:** 2016-06-29

**Authors:** Søren D. Østergaard, Janne T. Larsen, Søren Dalsgaard, Timothy E. Wilens, Preben B. Mortensen, Esben Agerbo, Ole Mors, Liselotte Petersen

**Affiliations:** 1 Department of Clinical Medicine, Aarhus University Hospital, Aarhus, Denmark; 2 Psychosis Research Unit, Aarhus University Hospital, Risskov, Denmark; 3 iPSYCH, The Lundbeck Foundation Initiative for Integrative Psychiatric Research, Aarhus, Denmark; 4 National Centre for Register-based Research, Aarhus University, Aarhus, Denmark; 5 Clinical and Research Programs in Pediatric Psychopharmacology, Massachusetts General Hospital, Harvard Medical School, Boston, MA, United States of America; 6 Department of Psychiatry, Harvard Medical School, Boston, MA, United States of America; 7 Centre for Integrated Register-based Research at Aarhus University (CIRRAU), Aarhus, Denmark; Western Sydney University, AUSTRALIA

## Abstract

**Background:**

Attention-deficit/hyperactivity disorder (ADHD) is a prevalent neurodevelopmental disorder with early onset. ADHD is associated with significant morbidity and mortality, partly due to delayed diagnosis. Identification of children at high risk for developing ADHD could lead to earlier diagnosis and potentially change the negative trajectory of the illness for the better. Since early psychosocial adversity is considered to be a likely etiological risk factor for ADHD, markers of this construct may be useful for early identification of children at high risk. Therefore, we sought to investigate whether Rutter’s indicators of adversity (low social class, severe marital discord, large family size, paternal criminality, maternal mental disorder, and placement in out-of-home care) assessed in infancy could serve as early predictors for the development of ADHD.

**Methods and Findings:**

Using data from the Danish nationwide population-based registers, we established a cohort consisting of all 994,407 children born in Denmark between January 1^st^ 1993 and December 31^st^ 2011 and extracted dichotomous values for the six Rutter’s indicators of adversity at age 0–12 months (infancy) for each cohort member. The cohort members were followed from their second birthday and the association between the sum of Rutter’s indicators of adversity (RIA-score) in infancy and subsequent development of ADHD was estimated by means of Cox regression. Also, the number needed to screen (NNS) to detect one case of ADHD based on the RIA-scores in infancy was calculated. During follow-up (9.6 million person-years), 15,857 males and 5,663 females from the cohort developed ADHD. For both males and females, there was a marked dose-response relationship between RIA-scores assessed in infancy and the risk for developing ADHD. The hazard ratios for ADHD were 11.0 (95%CI: 8.2–14.7) and 11.4 (95%CI: 7.1–18.3) respectively, for males and females with RIA-scores of 5–6, compared to males and females with RIA-scores of 0. Among males with RIA-scores of 5–6, 37.6% (95%CI: 27.0–50.7) had been diagnosed with ADHD prior to the age of 20, corresponding to a NNS of 3.0 (95%CI: 2.2–4.0).

**Conclusions:**

Rutter’s indicators of adversity assessed in infancy strongly predicted ADHD. This knowledge may be important for early identification of ADHD.

## Introduction

Attention-deficit/hyperactivity disorder (ADHD) is a prevalent neurodevelopmental disorder with early onset [1,2]. In a recent study it was shown that ADHD is associated with an increased risk of dying young, particularly among those diagnosed relatively late (at age 18 or older) [3]. Therefore, in order to change the trajectory for such individuals, and thereby potentially reducing excess mortality, earlier detection of ADHD should be a priority [4]. However, identifying ADHD at an early stage is challenging [5–7], particularly because the etiology of the disorder remains unclear [8,9]. If the etiology was known, assessment of etiological risk factors could lead to earlier diagnosis and identification of children at high risk for developing ADHD.

Early psychosocial adversity is considered to be a likely etiological risk factor for mental health problems, including ADHD [8,9]. This hypothesis stems from the landmark studies of mental disorder among ten-year-old children performed by Rutter and colleagues [10–12]. Through these studies, six familial/environmental characteristics associated with the development of mental disorder were identified: low social class, severe marital discord, large family size, paternal criminality, maternal mental disorder, and placement in foster care. These six characteristics are now widely known as Rutter’s indicators of adversity (RIA). It has been proposed that RIA tap into psychosocial adversity [13] and it has been demonstrated consistently across several populations that RIA are associated with mental disorder among children, in particular in the case of ADHD [13–16]. However, the vast majority of studies focusing on the association between RIA and ADHD are cross-sectional, i.e. the information regarding the indicators of adversity and ADHD diagnostic status are gathered simultaneously and at a fairly advanced age of the children, which leads to a substantial risk for reverse causality (e.g., ADHD in offspring leading to marital discord, or low income). Also, most of the prior studies are based on self-report of RIA, which introduces a risk for report bias. Furthermore, in most studies sample sizes have been modest. Therefore, the longitudinal association between RIA status in very early childhood and the risk of ADHD later in childhood, adolescence or early adulthood remains almost unknown. As a logical consequence, the potential value of the RIA as “predictors” for the development of ADHD is also unknown.

In order to test the predictive potential of RIA with regard to ADHD, we conducted a longitudinal study of a nationwide birth cohort using data on RIA and ADHD extracted from the Danish registers [17]. To minimize the degree of reverse causality, we assessed information regarding RIA in the first year of life (infancy) and tested for association between RIA in infancy, and the risk of developing ADHD in the following years. The main hypothesis of our study was that RIA assessed in infancy would be strongly associated with development of ADHD in childhood, adolescence or early adulthood.

## Methods

### Design

This is a population-based historical prospective cohort study. Data was obtained by register linkage via the unique personal registration numbers, which are assigned to all Danes at the time of birth or when obtaining an address in Denmark [18].

### Cohort and follow-up

A cohort consisting of all children born in Denmark between January 1^st^ 1993 and December 31^st^ 2011, who were living in Denmark at their 2^nd^ birthday, was established through the Danish Civil Registration System [18]. Mothers and fathers to all subjects in the cohort were identified through the same register. To rule out bias due to immigration, only children of parents who were both born in Denmark were included in the cohort. Since the ADHD diagnosis is very rarely given prior to the age of 2 in Danish psychiatry [19], we began follow-up on the cohort members’ second birthday and ended it at the occurrence of the first of the following events: a diagnosis of ADHD, emigration, death or December 31^st^ 2013.

### Primary outcome

The primary outcome of the study was the assignment of an ADHD-diagnosis following inpatient or outpatient contact at a psychiatric, neurological or pediatric department in Denmark, in the period between January 1^st^ 1995 and December 31^st^ 2013. Diagnostic data regarding ADHD was extracted from the Danish Psychiatric Central Research Register (DPCRR) [20] and from the Danish National Patient Register (DNPR) [21]. In these two registers, the 8^th^ International Classification of Diseases (ICD-8) [22] was used as diagnostic reference until January 1^st^ 1994, when it was replaced by the 10^th^ International Classification of Disease (ICD-10) [23]. Individuals receiving an ICD-10 diagnosis of F90.x or F98.8 following either inpatient or outpatient treatment were defined as ADHD cases in the present study. Since data from outpatients were only included in the DPCRR from January 1^st^ 1995, we began follow-up of the subjects on January 1^st^ 1995 at the earliest. To avoid bias due to the inclusion of cohort members with ADHD diagnosed prior to the initiation of follow-up, all individuals registered with a diagnosis of ADHD (defined by an ICD-10 diagnosis of F90.x or F98.8—or an ICD-8 diagnosis of 308.01) before their second birthday were excluded.

### Definition of Rutter’s indicators of adversity (RIA)

RIA were defined based on the nationwide Danish registers as described below. Due to the register-based nature of this study, we were unable to use the exact same definitions of RIA as derived by Rutter and colleagues [10–12] and those used by other groups [13–16]. Thus, the RIA defined below must be considered as approximations. The minor differences in the timing of the definition of the various RIA are due to differences in the timing of updates to the registers used in the study.

#### Low social class

Low social class of the parents of the subjects was defined as both parents being classified as “low” on at least one of the following variables: education, occupation or income. We defined low education as only having completed compulsory schooling (or not having completed compulsory schooling). A score of low regarding occupation was defined as receiving disability pension (in most cases this is a permanent pension due to mental and/or physical illness). Finally, low income was defined as an income in the lowest fifth of the general population for each sex and calendar year. This variable was based on data from the Danish Education Registers [24] the Integrated Database for Labor Market Research [25] and the Registers on Personal Income and Transfer Payments [25] For this variable, the parents’ educational and occupational statuses were assessed on October 1^st^ and November 1^st^, respectively, in the cohort members’ year of birth. Similarly, the parents’ gross income was obtained for the subject’s year of birth.

#### Severe marital discord

This variable was defined dichotomously according to whether both custodial parents were living at the same address as the subject or not on January 1^st^ in the year following the subject’s year of birth, based on information from Statistics Denmark’s FAIN dataset [26].

#### Large family size

Large family size was defined according to the number of children (<18 years of age) living in the household where the subject resided on January 1^st^ in the year after the cohort member’s year of birth. Large family was defined dichotomously as a household with four or more children (including the cohort member) [12]. This variable was based on information from Statistics Denmark’s FAFA dataset [27].

#### Paternal criminality

We defined paternal criminality dichotomously based on whether the father of the cohort member had ever received a custodial or suspended sentence for an offence under the principal Danish criminal acts (e.g., violence, robbery, arson, murder, sexual crime, theft, fraud, and extortion), the special legislation regarding drugs and weapons, or sections of the traffic act dealing with impaired driving. The data for this variable was extracted from the Danish National Crime Register [28] using information on all convictions since 1980 and defined based on paternal criminality prior to the cohort members’ 1^st^ birthday.

#### Maternal mental disorder

This variable was defined dichotomously based on whether the mother of the cohort member had ever been registered with a mental disorder (ICD-8 diagnoses: 290–315 and ICD-10 diagnoses: F00-F99) in the DPCRR. The variable was defined based on diagnoses registered from 1969 until the cohort members’ first birthday.

#### Placement in out-of-home care

This variable was defined dichotomously for each cohort member based on whether he or she had ever been placed outside home (in foster care or in an institution/orphanage) either with or without the parents’ consent. Data was extracted from the Register of Support for Children and Adolescents, which contains information on all placements made in Denmark since 1977 [29]. The variable was defined based on the status regarding placements registered prior to the cohort members’ first birthday.

### Statistical analyses

The data was analyzed by means of Cox regression using age as the underlying time-axis by means of the “stcox” command in Stata (version 13). Hazard ratios, Wald statistics, 95% confidence bands, and associated p-values were computed. All analyses were stratified by gender and adjusted for calendar year (1 year strata). The number needed to screen (NNS) was calculated as one divided by the difference between the risk of ADHD among cohort members with an increased RIA-score and those with a RIA-score equal to zero [30]. The risk of ADHD was estimated as one minus the Kaplan-Meier estimator.

Furthermore, to assess whether potential associations between elevated RIA-scores and ADHD were driven by large effects of single RIA, we conducted “leave-one-out” analyses in which each of the six RIA, in turn, were excluded from the RIA-score in the Cox regression analyses (carried out as described above) investigating the association between RIA-scores and ADHD (stratified by gender and adjusted for calendar year). Finally, we assessed the pair-wise association between the RIA by means of Pearson correlation.

### Autism spectrum disorder as control

In order to test the specificity of the association between RIA and ADHD, we included autism spectrum disorder (ASD) as “control” condition. ASD was chosen as control as the incidence pattern of this diagnostic category is that closest to ADHD among the major mental disorders [19]. Based on the comorbidity between ASD and ADHD [31–33], and the general overlap in risk factors for mental disorders [34,35], we were expecting to find some degree of association between RIA and ASD, but weaker than that for RIA and ADHD. The same birth cohort as described above for ADHD was used for the analysis of the association between RIA and ASD. The methods applied for the analysis of ASD (analogue to those used for the analysis of ADHD) are described in detail in S1 Text.

### Ethics

The use of data from the registers was approved by the Danish Data Protection Agency, the Danish National Board of Health and Statistics Denmark.

## Results

We identified 1,000,296 children born between January 1^st^ 1993 and December 31^st^ 2011 to Danish born parents. Of these, 5,889 either died (n = 4,494), emigrated / were lost to follow-up (n = 1,195), or received an ADHD diagnosis (n = 200) before their second birthday. Thus, 994,407 children (510,213 males and 484,194 females) were followed from their 2-year birthday yielding a total of 9,620,404 person-years of observation. The prevalence of the six RIA in infancy were as follows in this cohort: paternal criminality = 10.7%, severe marital discord = 9.9%, low social class = 9.1%, maternal mental disorder = 5.5%, large family size = 3.5%, placement in out-of-home care = 0.3%.

**Fig 1:**
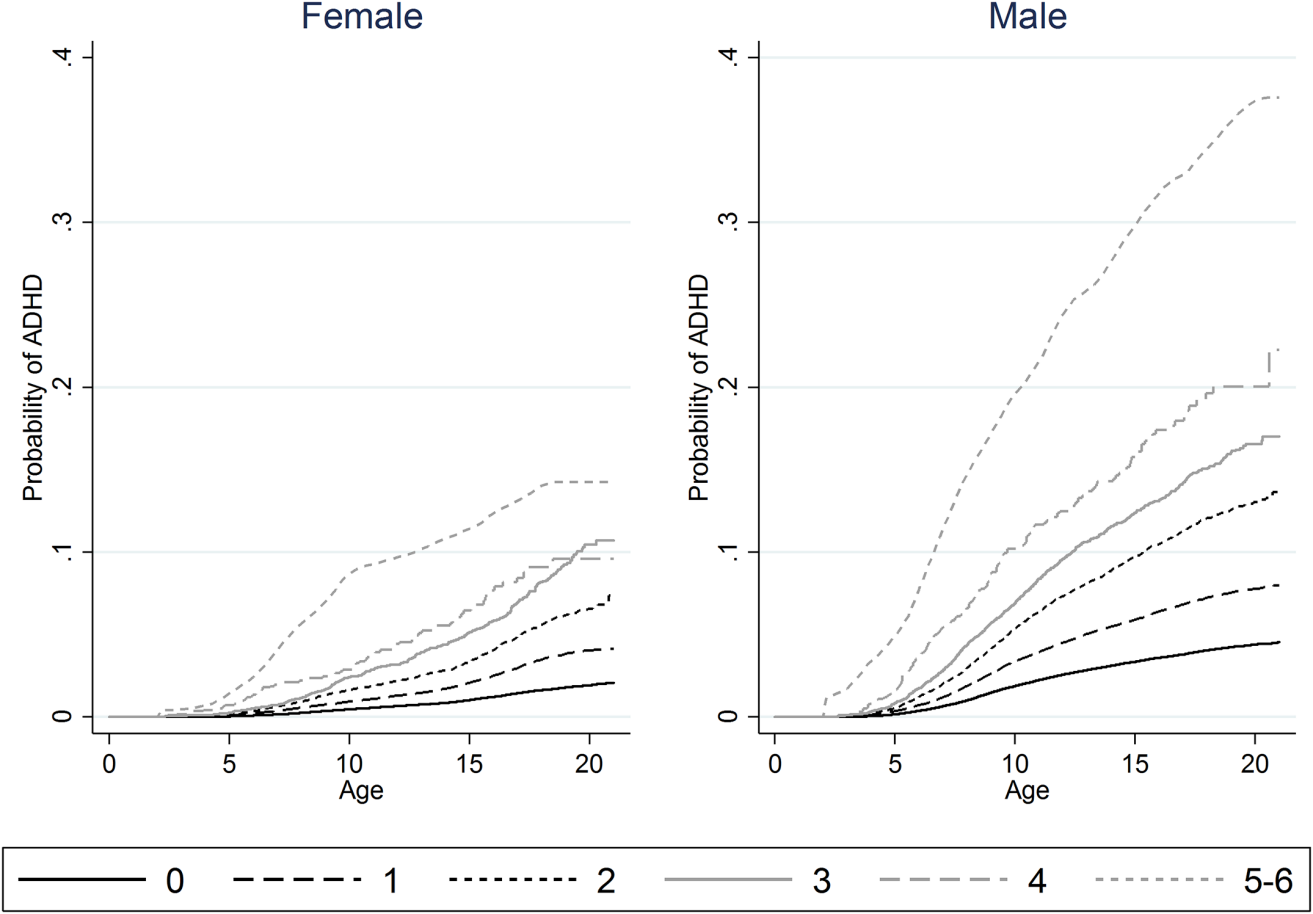
Proportion of cohort members developing ADHD over time stratified by RIA-score. Kaplan-Meier plots showing the proportion of the female (left) and male (right) cohort members developing ADHD over time stratified by RIA-score in infancy (as represented by the curves). Due to the relatively low number of cohort members with 5–6 RIA, these curves were smoothened (local polynomial kernel smoothing (Epanechnikov)) to avoid potential identification of individuals. Furthermore, to remain within the boundaries of the data, the probability of ADHD was manually restricted to zero until the age of 2 years (the beginning of follow-up) for the two curves representing individuals with RIA-scores of 5–6.

During follow-up, 15,857 males and 5,663 females from the cohort were diagnosed with ADHD, corresponding to incidence rates of 3.2 (95%CI: 3.2–3.3) and 1.2 (95%CI: 1.2–1.2) per 1,000 person-years for males and females respectively. Table 1 shows incidence rates and hazard ratios for the development of ADHD for each of the six RIA, as well as for the summed RIA, stratified on males and females. The incidence rates for ADHD per 1,000 person-years for the six RIA ranged from 3.1 (95%CI: 2.9–3.4) and 1.2 (95%CI: 1.1–1.2) for large family size to 17.6 (95%CI: 15.3–20.2) and 8.6 (95%CI: 7.0–10.5) for placement in out-of-home care for males and females respectively. The corresponding hazard ratios ranged from 1.0 (95%CI: 0.9–1.1) and 1.0 (95%CI: 0.9–1.2) to 5.6 (95%CI: 4.9–6.5) and 7.4 (95%CI: 6.1–9.1). Furthermore, there was a strong dose-response relationship between the sum of RIA (RIA-score) and the risk for subsequent ADHD as also visualized by the Kaplan-Meier plots (Fig 1).

**Table 1:** Incidence rates and adjusted hazard ratios for ADHD for each of Rutter’s indicators of adversity (RIA) and for the total RIA-score assessed in infancy.

FEMALES	N ADHD (%)	N total(%)	Rate (95% CI)	HR (95% CI)
**Low social class**				
**No**	4,240 (74.87)	439,782 (90.83)	1.00 (0.97–1.03)	1.00 (ref)
**Yes**	1,416 (25.00)	44,082 (9.10)	2.95 (2.80–3.10)	2.94 (2.77–3.12)
**Severe marital discord**				
**No**	4,425 (78.14)	435,379 (89.92)	1.05 (1.02–1.08)	1.00 (ref)
**Yes**	1,228 (21.68)	47,794 (9.87)	2.47 (2.34–2.62)	2.34 (2.20–2.49)
**Large family size**				
**No**	5,450 (96.24)	466,038 (96.25)	1.20 (1.17–1.23)	1.00 (ref)
**Yes**	203 (3.58)	17,135 (3.54)	1.22 (1.07–1.40)	1.02 (0.88–1.17)
**Paternal criminality**				
**No**	4,367 (77.11)	432,540 (89.33)	1.04 (1.01–1.07)	1.00 (ref)
**Yes**	1,296 (22.89)	51,654 (10.67)	2.59 (2.45–2.73)	2.50 (2.35–2.66)
**Maternal mental disorder**				
**No**	5,164 (91.19)	457,738 (94.54)	1.14 (1.11–1.17)	1.00 (ref)
**Yes**	499 (8.81)	26,456 (5.46)	2.72 (2.50–2.97)	2.56 (2.34–2.81)
**Out-of-home care**				
**No**	5,565 (98.27)	482,936 (99.74)	1.18 (1.15–1.22)	1.00 (ref)
**Yes**	98 (1.73)	1,258 (0.26)	8.58 (7.04–10.46)	7.43 (6.08–9.07)
**RIA-score**				
**0**	2,775 (49.00)	348,594 (71.99)	0.82 (0.79–0.85)	1.00 (ref)
**1**	1,615 (28.52)	96,140 (19.86)	1.72 (1.64–1.80)	2.10 (1.97–2.23)
**2**	806 (14.23)	28,395 (5.86)	2.86 (2.67–3.07)	3.50 (3.24–3.79)
**3**	372 (6.57)	9,024 (1.86)	4.25 (3.84–4.71)	5.25 (4.71–5.85)
**4**	78 (1.38)	1,829 (0.38)	4.93 (3.95–6.15)	6.14 (4.90–7.69)
**5–6**	17 (0.30)	212 (0.04)	9.10 (5.65–14.63)	11.37 (7.06–18.32)
**MALES**				
**Low social class**				
**No**	12,355 (77.92)	463,189 (90.78)	2.80 (2.75–2.85)	1.00 (ref)
**Yes**	3,491 (22.02)	46,655 (9.14)	7.02 (6.80–7.26)	2.61 (2.51–2.71)
**Severe marital discord**				
**No**	12,764 (80.49)	458,488 (89.86)	2.91 (2.86–2.96)	1.00 (ref)
**Yes**	3,073 (19.38)	50,707 (9.94)	5.95 (5.74–6.17)	2.07 (1.99–2.15)
**Large family size**				
**No**	15,292 (96.44)	491,126 (96.26)	3.23 (3.18–3.29)	1.00 (ref)
**Yes**	545 (3.44)	18,069 (3.54)	3.13 (2.88–3.40)	0.96 (0.89–1.05)
**Paternal criminality**				
**No**	12,481 (78.71)	456,072 (89.39)	2.84 (2.79–2.89)	1.00 (ref)
**Yes**	3,376 (21.29)	54,141 (10.61)	6.58 (6.36–6.80)	2.33 (2.25–2.42)
**Maternal mental disorder**				
**No**	14,612 (92.15)	482,564 (94.58)	3.09 (3.04–3.14)	1.00 (ref)
**Yes**	1,245 (7.85)	27,649 (5.42)	6.62 (6.26–7.00)	2.15 (2.03–2.28)
**Out-of-home care**				
**No**	15,663 (98.78)	508,951 (99.75)	3.20 (3.15–3.25)	1.00 (ref)
**Yes**	194 (1.22)	1,262 (0.25)	17.55 (15.25–20.20)	5.60 (4.86–6.45)
**RIA-score**				
**0**	8,451 (53.30)	367,079 (71.95)	2.39 (2.34–2.44)	1.00 (ref)
**1**	4,254 (26.83)	101,784 (19.95)	4.31 (4.19–4.44)	1.82 (1.76–1.89)
**2**	2,072 (13.07)	29,710 (5.82)	7.22 (6.91–7.54)	3.08 (2.93–3.23)
**3**	841 (5.30)	9,511 (1.86)	9.39 (8.77–10.04)	4.02 (3.75–4.32)
**4**	193 (1.22)	1,902 (0.37)	12.46 (10.82–14.35)	5.27 (4.57–6.08)
**5–6**	46 (0.29)	227 (0.04)	25.99 (19.46–34.69)	10.99 (8.22–14.68)

”N ADHD” refers to the number of ADHD cases in each stratum, while”N total” is the total number of individuals in each stratum. The rates are per 1,000 person-years. The hazard ratios (HR) are adjusted for calendar year (1 year strata). Information regarding low social class, severe marital discord and large family size was unavailable for 0.08%, 0.14%, and 0.14% of the ADHD cases respectively. For the entire cohort, the corresponding proportions were 0.07%, 0.21% and 0.21%.

Table 2 shows the NNS for detecting ADHD prior to the age of 5, 10, 15 and 20 years respectively, based on RIA assessed in infancy. The NNS values were lower for males than for females, and the values decreased with age and increasing number of RIA. Notably, 37.6% (95%CI: 27.0–50.7) of the male and 14.1% (95%CI: 8.4–23.2) of the females cohort members with RIA-scores of 5–6 had been diagnosed ADHD prior to the age of 20 years, corresponding to NNS of 3.0 (95%CI: 2.2–4.0) and 8.2 (95%CI: 4.7–15.3) respectively.

**Table 2:** The number needed to screen to detect one case of ADHD based on the Rutter’s Indicators of adversity score (RIA-score) assessed in infancy.

	Number needed to screen (95% CI)			
	Prior to age 5	Prior to age 10	Prior to age 15	Prior to age 20
**FEMALES**				
**RIA-score**				
**1**	1,460.43 (1,171.88–1,823.03)	204.69 (186.54–224.77)	94.30 (87.70–101.45)	47.23 (44.11–50.58)
** 2**	942.21 (649.51–1,388.17)	84.09 (74.36–95.22)	42.98 (38.91–47.54)	21.64 (19.68–23.81)
**3**	484.20 (293.07–819.91)	50.60 (42.34–60.68)	24.34 (21.20–28.01)	11.74 (10.24–13.49)
**4**	143.92 (80.01–262.49)	41.36 (28.96–60.13)	18.45 (13.91–24.79)	13.05 (9.72–17.83)
**5–6**	64.90 (20.81–211.79)	12.52 (7.29–22.12)	9.30 (5.59–16.13)	8.22 (4.74–15.25)
**MALES**				
**RIA-score**				
**1**	517.66 (455.10–589.44)	66.64 (63.24–70.25)	39.08 (37.42–40.84)	29.75 (28.44–31.13)
**2**	276.35 (226.26–339.14)	28.81 (26.83–30.97)	15.78 (14.92–16.71)	11.57 (10.91–12.28)
**3**	145.78 (112.17–190.79)	19.86 (17.83–22.17)	11.06 (10.15–12.07)	8.22 (7.51–9.02)
**4**	68.21 (45.58–103.37)	12.00 (9.92–14.63)	8.08 (6.78–9.69)	6.38 (5.32–7.74)
**5–6**	18.99 (10.59–34.69)	5.95 (4.24–8.55)	3.60 (2.71–4.92)	3.02 (2.17–4.41)

The number needed to screen was calculated as one divided by the difference between the risk of ADHD among cohort members with an increased RIA-score and those with a RIA-score equal to zero [30]. The risk of ADHD was estimated as one minus the Kaplan-Meier estimator.

The results of the leave-one-out analyses are shown in Table 3. The hazard ratios generated by these analyses show that the dose-response relationship between the RIA-score and the risk for ADHD is maintained irrespective of which individual RIA is excluded from the score.

**Table 3:** Leave-one-out analyses of the association between the Rutter’s Indicators of adversity score (RIA-score) assessed in infancy and ADHD.

	FEMALES	MALES
	HR (95% CI)	HR (95% CI)
**RIA-score excl. low social class**		
**0**	1.00 (ref)	1.00 (ref)
**1**	2.03 (1.91–2.15)	1,81 (1,75–1,88)
**2**	3.59 (3.29–3.92)	3,03 (2,87–3,21)
**3**	5.42 (4.54–6.47)	4,55 (4,06–5,09)
**4**	8.96 (5.94–13.50)	7,84 (6,04–10,17)
**5**	N/A	22,84 (5,71–91,33)
**RIA-score excl. severe marital discord**		
**0**	1.00 (ref)	1.00 (ref)
**1**	2.27 (2.14–2.41)	2.07 (1.99–2.14)
**2**	3.91 (3.60–4.25)	3.31 (3.15–3.49)
**3**	4.77 (3.99–5.71)	3.89 (3.47–4.36)
**4**	11.66 (7.98–17.03)	8.80 (6.70–11.56)
**5**	N/A	13.60 (1.92–96.57)
**RIA-score excl. large family size**		
**0**	1.00 (ref)	1.00 (ref)
**1**	2.22 (2.09–2.36)	1.94 (1.87–2.02)
**2**	3.75 (3.47–4.06)	3.22 (3.07–3.39)
**3**	5.35 (4.78–5.99)	4.37 (4.06–4.69)
**4**	7.63 (6.00–9.70)	6.40 (5.49–7.45)
**5**	14.10 (8.49–23.42)	10.53 (7.44–14.90)
**RIA-score excl. paternal criminality**		
**0**	1.00 (ref)	1.00 (ref)
**1**	2.14 (2.02–2.27)	1.89 (1.82–1.96)
**2**	3.85 (3.54–4.19)	3.13 (2.97–3.30)
**3**	5.76 (4.86–6.82)	4.38 (3.91–4.91)
**4**	8.66 (5.69–13.17)	9.08 (7.14–11.54)
**5**	N/A	13.33 (1.88–94.61)
**RIA-score excl. maternal mental disorder**		
**0**	1.00 (ref)	1.00 (ref)
**1**	2.14 (2.01–2.27)	1.81 (1.74–1.87)
**2**	3.48 (3.21–3.77)	3.13 (2.98–3.29)
**3**	4.96 (4.41–5.58)	4.00 (3.71–4.32)
**4**	7.61 (5.70–10.15)	6.41 (5.33–7.72)
**5**	N/A	9.20 (2.30–36.78)
**RIA-score excl. out-of-home care**		
**0**	1.00 (ref)	1.00 (ref)
**1**	2.10 (1.98–2.24)	1.82 (1.76–1.89)
**2**	3.55 (3.29–3.84)	3.12 (2.98–3.27)
**3**	5.43 (4.88–6.04)	4.05 (3.78–4.35)
**4**	4.72 (3.59–6.21)	4.97 (4.24–5.83)
**5**	5.30 (1.33–21.22)	12.29 (7.13–21.17)

In the “leave-one-out” analyses each of the six RIA were excluded, in turn, from the Cox regression analyses investigating the association between RIA-scores and ADHD (stratified by gender and adjusted for calendar year). N/A = The number of individuals in these cells were too low to allow for analysis.

The pair-wise association between RIA is shown in Table 4. The Pearson correlation coefficients ranged from -0.0059 for out-of-home care and large family size to 0.2417 for paternal criminality and low social class.

**Table 4:** The pairwise association between Rutter’s Indicators of adversity assessed in infancy.

	Low social class	Severe marital discord	Large family size	Paternal criminality	Maternal mental disorder	Out-of-home care
**Low social class**	1.0000					
**Severe marital discord**	0.2074	1.0000				
**Large family size**	0.0502	0.0045	1.0000			
**Paternal criminality**	0.2417	0.1854	0.0272	1.0000		
**Maternal mental disorder**	0.0858	0.0860	0.0071	0.0730	1.0000	
**Out-of-home care**	0.0959	0.1176	-0.0059	0.0616	0.0753	1.0000

The pairwise association between the individual Rutter’s Indicators of adversity was assessed by means of Pearson correlation. This table contains the resulting Pearson correlation coefficients.

The results of the analysis of the association between RIA and ASD are reported in S1 Text, S1, S2, and S3 Tables respectively. In brief, the hazard ratios for ASD ranged from 0.9 (95%CI: 0.8–1.0) and 0.8 (95%CI: 0.7–1.0) for large family size to 3.5 (95%CI: 2.9–4.4) and 4.2 (95%CI: 2.9–6.0) for placement in out-of-home care for males and females respectively (S1 Table). A total of 17.1% (95%CI: 10.0–28.6) of the male and 2.1% (95%CI: 0.8–5.6) of the females cohort members with RIA-scores of 5–6 had been diagnosed ASD prior to the age of 20 years, corresponding to NNS of 7.1 (95%CI: 4.0–14.5) and 101.5 (95%CI: 22.5–344.0) respectively (S2 Table). When adjusting for ADHD, the corresponding NNS were 9.3 (95%CI: 4.3–26.0) and 157.3 (95%CI: 24.3–220.0) respectively (S3 Table).

## Discussion

In this study of 994,407 children followed for more than 9.6 million person-years, we tested the association between Rutter’s indicators of adversity (RIA) score in infancy assessed via nationwide registers, and the risk for developing ADHD later in childhood/adolescence/early adulthood. The main finding was that the risk of ADHD increased in a dose-response like manner with increasing RIA load. This is consistent with findings from prior studies of smaller samples, where RIA were assessed later in childhood [13–16].

As opposed to the five other RIA, large family size was not significantly associated with subsequent ADHD. In the early 1970’s, when the studies establishing RIA as risk factors for mental illness in children were conducted in the United Kingdom, having a large family was indicative of low socioeconomic status [10–12]. This relationship is probably less pronounced in Denmark in the period studied in this analysis, during which the government subsidized families with children (per child) with a yearly amount until the child turned 18 years old. The amount depends on the income of the parents, and is currently up to 1,900 USD per year on average (more in the younger years and gradually less as the children age). In contrast, the RIA with the strongest predictive ability in the present study was placement in out-of-home care, a finding, which is in accordance with previous studies suggesting a strong association between early institutionalization and ADHD [36–39].

Despite the fact that our results show that there are substantial differences in the predictive ability of the various RIA, the maintained dose-response relationship across the leave-one-out analyses indicate that the RIA-ADHD association is not driven by a single indicator—and that it is reasonable to use the sum of the RIA as an overall measure of adversity. In further support of this practice, the relatively low values for the Pearson correlation coefficients stemming from the analysis of the pair-wise association of the RIA (Table 4) indicate that the RIA tap into different aspects of adversity. As an example, the largest correlation coefficient of 0.2417, which was found for paternal criminality and low social class, implies that less than 6% (the squared correlation = 0.2417^2^ = 0.058) of the variance in paternal criminality is accounted for by low social class and vice versa.

While early assessment of RIA in itself, as indicated by the low values for NNS, appear to be quite sensitive in identifying infants at risk for ADHD, particularly among males, the predictive ability is likely to be even higher for a composite “test-battery”. For instance, combining RIA assessment with biomarkers [40], evaluation of cognitive function and motor skills [7,41] and a clinical assessment of early ADHD- and related symptoms [7,41,42], may result in even lower NNS. If adding the increased prior probability of ADHD conveyed by parents’/nurses’/physicians’ suspicion of early developmental abnormalities, such a test-battery may be sufficiently predictive to be implemented in clinical practice to identify infants at high risk for ADHD. This perspective should be subjected to further investigation.

Since the cohort studied here overlaps substantially with the one used in the recent study of mortality by Dalsgaard et al. [3], it seems possible that our findings could potentially have implications for the mortality associated with ADHD as well. Dalsgaard et al. demonstrated that being diagnosed with ADHD at the age of 6 years or older (particularly for those diagnosed at 18 years or older) was associated with significantly increased mortality, whereas being diagnosed at the age of 5 years or younger was not [3]. This could entail that lowering the average age at diagnosis of ADHD, for instance by means of identifying high-risk populations through assessment of RIA, could change the disease trajectory for individuals with ADHD, and maybe, in turn, reduce the increased mortality associated with the disorder [4].

From an etiological perspective, there is little doubt that RIA tap into both genetic and environmental causal mechanisms for ADHD, as well as interactions between the two [43]. However, based on the data used in this observational study, we were not able to disentangle genetic and environmental effects, nor can we infer causality. Nevertheless, judged by the strong associations between RIA and ADHD reported here, future studies with access to data regarding genetic variants and (environmental) RIA data, as well as ADHD-status, may offer important insights into the etiology of ADHD [8,9,43].

In order to study the specificity of the RIA-ADHD association, we conducted analogue analyses of the association between RIA assessed in infancy and subsequent development of autism spectrum disorder (ASD). The results of these analysis showed, that while there were positive associations (hazard ratios above 1) between the RIA and ASD (apart from for large family size), the strength of these associations appears weaker than for those reported for RIA and ADHD (compare Table 2 with S1 Table). Also, the associations were weakened further when adjusted for ADHD, indicating that they are partly driven by comorbid ADHD. In consideration of the major symptomatological overlap between ASD and ADHD (the proportion of children with ASD that have significant symptoms of hyperactivity/impulsivity and/or inattention has been reported to be within the range of 31–95% [31–33]), the degree of specificity of the RIA-ADHD association found in this study is more pronounced than we expected. Furthermore, the fact that the RIA-ADHD association is not completely specific does not rule out its potential value in early identification of ADHD, as a high RIA-score would never be enough to assign a diagnosis of ADHD. The latter would rely on a standard diagnostic assessment, during which alternative diagnoses such as ASD would also be considered.

The most important limitation of our study is the use of register-based approximations of RIA. While our definitions of low social class, large family size, paternal criminality, maternal mental disorder, and *placement in out-of-home care* are quite similar to those used in other studies, the definition of *severe marital discord* differs more substantially. Since the registers do not contain information about the degree of conflicts among cohabiting individuals, we operationalized this particular variable dichotomously according to whether both custodial parents were living at the same address as the infant or not. Based on the present data, we are unable to determine whether this definition captures the same construct as that originally defined by Rutter and colleagues [10–12]. However, this is little different from other studies of RIA and ADHD [13–16], which reveal that there is currently no consensus regarding the definition of severe marital discord.

A related limitation of the study is the use of a register-based definition of ADHD. The diagnoses in the registers providing data for this study are clinical diagnoses, and are not necessarily assigned based on standardized research interviews as in most clinical studies. However, the validity of the clinical ADHD diagnoses in the registers is high [44,45]. The register-based definition of ADHD also has implications for the generalizability of our results since private practicing pediatricians, child and adolescent psychiatrists, and psychiatrists, who diagnose and treat a minority of ADHD cases in Denmark, do not report to the registers. Furthermore, individuals in whom ADHD has not been identified are not classified as “cases” in this study. Therefore, our results are probably most valid for the more severe cases within the ADHD spectrum. Finally, the register-based nature of this study entails that translation of the results into clinical practice is not straightforward. Therefore, the predictive ability of early assessment of RIA, ideally in combination with genetic or other biological markers, should be investigated in prospective clinical studies.

The register-based approach of this study also has important strengths. First of all, the sample size of 994,407 makes this the far largest study of the RIA-ADHD association ever conducted. Secondly, while most previous studies testing the association between RIA and ADHD [13–16] have relied on cross-sectional data, often with self-report regarding RIA, the present study is based on longitudinal data from registers (not self-reported), which practically eliminates the risk of report bias and reduces the risk of reverse causality. However, the risk for reverse causality is by no means eliminated as some degree of case-finding bias is likely to affect the associations reported here. For example, children placed in out-of-home care may be more likely to be evaluated for potential ADHD as an intrinsic part of the assessment prior to placement in out-of-home care. Furthermore, it cannot be excluded that very early and severe ADHD symptoms may affect RIA, i.e. increase the risk of maternal mental disorder, severe marital discord or placement in out-of-home care. However, the extent to which such reverse causation has occurred cannot be determined based on the data at hand.

In terms of generalizability, it is important to note that Denmark is among the most economically and socially equal welfare states in the world [46], and the strong association between RIA assessed in infancy and ADHD documented in this study may therefore not be representative for societies providing other levels of welfare to its citizens. However, if RIA-ADHD associations of similar strength exist in less egalitarian and more unequal societies, the ADHD-predictive potential of the RIA will be even more pronounced from a public health perspective, under the assumption that a relatively larger proportion of children will be growing up under psychosocially adverse circumstances (high RIA-scores).

Although it is still too early to make clinical recommendations based on associations of RIA and ADHD, this study is the first to show that assessment of RIA in infants may be useful in identifying individuals at high risk of developing ADHD later in childhood or adolescence. Ideally, this possibility should be subjected to prospective clinical investigation. Furthermore, although no causal inference can be made at this point, the strong dose-dependent association between RIA and ADHD, may imply that RIA represent etiological factors for ADHD. Since the etiology of ADHD remains unclear, this hypothesis seems worth pursuing in further studies.

## Supporting Information

S1 Checklist (PDF)

S1 Table Incidence rates and adjusted hazard ratios for ASD for each of Rutter’s indicators of adversity (RIA) and for the total RIA-score assessed in infancy. ”N ASD” refers to the number of ASD cases in each stratum, while”N total” is the total number of individuals in each stratum. The rates are per 1,000 person-years. The hazard ratios (HR) are adjusted for calendar year (1 year strata). Information regarding low social class, severe marital discord and large family size was unavailable for 0.03%, 0.16%, and 0.16% of the ASD cases respectively. For the entire cohort, the corresponding proportions were 0.07%, 0.21% and 0.21%.
(DOCX)

S2 Table The number needed to screen to detect one case of ASD based on the Rutter’s Indicators of adversity score (RIA-score) assessed in infancy. The number needed to screen was calculated as one divided by the difference between the risk of ADHD among cohort members with an increased RIA-score and those with a RIA-score equal to zero [30]. The risk of ASD was estimated as one minus the Kaplan-Meier estimator.
(DOCX)

S3 Table ADHD-adjusted number needed to screen to detect one case of ASD based on the Rutter’s Indicators of adversity score (RIA-score) assessed in infancy. The number needed to screen was calculated as one divided by the difference between the risk of ASD among cohort members with an increased RIA-score and those with a RIA-score equal to zero [30]. The risk of ASD was estimated as one minus the Kaplan-Meier estimator. In this analysis (compared to that providing the results described in S2 Table), cohort members registered with a diagnosis of ADHD prior to initiation of follow-up for ASD were excluded. Furthermore, receiving a diagnosis of ADHD was included among the events censoring follow-up for ASD.
(DOCX)

S1 Text (DOCX)

## Abbreviations

ADHD: Attention-deficit/hyperactivity disorder
RIA: Rutter’s Indicators of adversity
NNS: Number needed to screen
ASD: Autism spectrum disorder
