# Supplementary material for: Predicting ADHD by Assessment of Rutter’s Indicators of Adversity in Infancy
Source: PLoS One. 2016 Jun 29;11(6):e0157352. doi: 10.1371/journal.pone.0157352 (PMC4927115; doi:10.1371/journal.pone.0157352)
Supplement: S1 Checklist — (PDF) [file pone.0157352.s001.pdf]

STROBE Statement—checklist of items that should be included in reports of observational studies

	Item No	Recommendation
**Title and abstract**	1	(*a*) Indicate the study's design with a commonly used term in the title or the abstract<br>Done: "Using data from the Danish nationwide population-based registers, we established a cohort consisting of all 994,407 children born in Denmark between January 1st 1993 and December 31st 2011 and extracted dichotomous values for the six Rutter's indicators of adversity at age 0-12 months (infancy) for each cohort member. The cohort members were followed from their second birthday and the association between the sum of Rutter's indicators of adversity (RIA-score) in infancy and subsequent development of ADHD was estimated by means of Cox regression."
		(*b*) Provide in the abstract an informative and balanced summary of what was done and what was found<br>Done: "During follow-up (9.6 million person-years), 15,857 males and 5,663 females from the cohort developed ADHD. For both males and females, there was a marked dose-response relationship between RIA-scores at infancy and the risk for developing ADHD. The hazard ratios for ADHD were 11.0 (95%CI: 8.2-14.7) and 11.4 (95%CI: 7.1-18.3) respectively, for males and females with RIA-scores of 5-6, compared to males and females with RIA-scores of 0. Among males with RIA-scores of 5-6, 37.6% (95%CI: 27.0-50.7) had been diagnosed with ADHD at age 20, corresponding to a NNS of 3.0 (95%CI: 2.2-4.0)."
**Introduction**		
Background/rationale	2	Explain the scientific background and rationale for the investigation being reported<br>Done: "It has been proposed that RIA tap into psychosocial adversity [13] and it has been demonstrated consistently across several populations that RIA are associated with mental disorder among children, in particular in the case of ADHD [13-16]. However, the vast majority of studies focusing on the association between RIA and ADHD are cross-sectional, i.e. the information regarding the indicators of adversity and ADHD diagnostic status are gathered simultaneously and at a fairly advanced age of the children, which leads to a substantial risk for reverse causality (e.g., ADHD in offspring leading to marital discord, or low income). Also, most of the prior studies are based on self-report of RIA, which introduces a risk for report bias. Furthermore, in most studies sample sizes have been modest. Therefore, the longitudinal association between RIA status in very early childhood and the risk of ADHD later in childhood, adolescence or early adulthood remains almost unknown"
Objectives	3	State specific objectives, including any prespecified hypotheses<br>Done: "As a logical consequence, the potential value of the RIA as "predictors" for the development of ADHD is also unknown. This led us to conduct a longitudinal study of a nationwide birth cohort using data on RIA and ADHD extracted from the Danish registers"
**Methods**		
Study design	4	Present key elements of study design early in the paper<br>Done: "This is a population-based historical prospective cohort study. Data was obtained by register linkage via the unique personal registration numbers, which are assigned to all Danes at the time of birth or when obtaining an address in Denmark [18]."
Setting	5	Describe the setting, locations, and relevant dates, including periods of recruitment, exposure, follow-up, and data collection<br>Done: See below.
Participants	6	*Cohort study*—Give the eligibility criteria, and the sources and methods of selection of participants. Describe methods of follow-up<br>*Done: "*A cohort consisting of all children born in Denmark between January 1st 1993 and December 31st 2011, who were living in Denmark at their 2nd birthday, was established through the Danish Civil Registration System [18]."

Variables	7	Clearly define all outcomes, exposures, predictors, potential confounders, and effect modifiers. Give diagnostic criteria, if applicable<br>Done: All this information is given in the methods section.
Data sources/ measurement	8*	For each variable of interest, give sources of data and details of methods of assessment (measurement). Describe comparability of assessment methods if there is more than one group<br>Done: See the paragraph ”Definition of Rutter's indicators of adversity (RIA)” in the method section of the paper.
Bias	9	Describe any efforts to address potential sources of bias<br>Done: “However, the vast majority of studies focusing on the association between RIA and ADHD are cross-sectional, i.e. the information regarding the indicators of adversity and ADHD diagnostic status are gathered simultaneously and at a fairly advanced age of the children, which leads to a substantial risk for reverse causality (e.g., ADHD in offspring leading to marital discord, or low income). Also, most of the prior studies are based on self-report of RIA, which introduces a risk for report bias.”
Study size	10	Explain how the study size was arrived at<br>Done: “We identified 1,000,296 children born between January 1st 1993 and December 31st 2011 to Danish born parents. Of these, 5,889 either died (n=4,494), emigrated / were lost to follow-up (n=1,195), or received an ADHD diagnosis (n=200) before their second birthday. Thus, 994,407 children (510,213 males and 484,194 females) were followed from their 2-year birthday yielding a total of 9,620,404 person-years of observation.”
Quantitative variables	11	Explain how quantitative variables were handled in the analyses. If applicable, describe which groupings were chosen and why<br>Done: See the section ”Definition of Rutter's indicators of adversity (RIA)” in the method section of the paper.
Statistical methods	12	(*a*) Describe all statistical methods, including those used to control for confounding<br>Done: “The data was analyzed by means of Cox regression using age as the underlying time-axis by means of the “stcox” command in Stata (version 13). Hazard ratios, Wald statistics, 95% confidence bands, and associated p-values were computed. All analyses were stratified by gender and adjusted for calendar year (1 year strata). The number needed to screen (NNS) was calculated as one divided by the difference between the risk of ADHD among cohort members with an increased RIA-score and those with a RIA-score equal to zero [30]. The risk of ADHD was estimated as one minus the Kaplan-Meier estimator.”
		(*b*) Describe any methods used to examine subgroups and interactions<br>Done: See method section.
		(*c*) Explain how missing data were addressed<br>Done: “We identified 1,000,296 children born between January 1st 1993 and December 31st 2011 to Danish born parents. Of these, 5,889 either died (n=4,494), emigrated / were lost to follow-up (n=1,195), or received an ADHD diagnosis (n=200) before their second birthday.”
		(*d*) *Cohort study*—If applicable, explain how loss to follow-up was addressed<br>Done: “See above”
		(*e*) Describe any sensitivity analyses<br>Done: See the description of the results displayed in S1 Table.

**Results**		
Participants	13*	(a) Report numbers of individuals at each stage of study—eg numbers potentially eligible, examined for eligibility, confirmed eligible, included in the study, completing follow-up, and analysed<br>Done: "We identified 1,000,296 children born between January 1st 1993 and December 31st 2011 to Danish born parents. Of these, 5,889 either died (n=4,494), emigrated / were lost to follow-up (n=1,195), or received an ADHD diagnosis (n=200) before their second birthday. Thus, 994,407 children (510,213 males and 484,194 females) were followed from their 2-year birthday yielding a total of 9,620,404 person-years of observation."
		(b) Give reasons for non-participation at each stage<br>Done: See above.
		(c) Consider use of a flow diagram<br>Done: Not considered necessary in this case
Descriptive data	14*	(a) Give characteristics of study participants (eg demographic, clinical, social) and information on exposures and potential confounders<br>Done: See Table 1
		(b) Indicate number of participants with missing data for each variable of interest<br>Done: See Table 1
		(c) Cohort study—Summarise follow-up time (eg, average and total amount)<br>Done: "Thus, 994,407 children (510,213 males and 484,194 females) were followed from their 2-year birthday yielding a total of 9,620,404 person-years of observation."
Outcome data	15*	*Cohort study*—Report numbers of outcome events or summary measures over time
		Case-control study—Report numbers in each exposure category, or summary measures of exposure<br>n/a
		Cross-sectional study—Report numbers of outcome events or summary measures<br>n/a
Main results	16	*(*a) Give unadjusted estimates and, if applicable, confounder-adjusted estimates and their precision (eg, 95% confidence interval). Make clear which confounders were adjusted for and why they were included<br>Done: See Table 1
		*(*b) Report category boundaries when continuous variables were categorized<br>Done: See the paragraph "Definition of Rutter's indicators of adversity (RIA)" in the method section of the paper.
		*(*c) If relevant, consider translating estimates of relative risk into absolute risk for a meaningful time period<br>Done: See Table 2
Other analyses	17	Report other analyses done—eg analyses of subgroups and interactions, and sensitivity analyses<br>Done: See the description of the results displayed in S1 Table.
**Discussion**		
Key results	18	Summarise key results with reference to study objectives<br>Done: "In this study of 994,407 children followed for more than 9.6 million person-years, we tested the association between Rutter's indicators of adversity (RIA) score in infancy assessed via nationwide registers, and the risk for developing ADHD later in childhood/adolescence/early adulthood. The main finding was that the risk of ADHD increased in a dose-response like manner with increasing RIA load. This is consistent with findings from prior studies of smaller samples, where RIA were assessed later in childhood [13-16]."

Limitations	19	Discuss limitations of the study, taking into account sources of potential bias or imprecision. Discuss both direction and magnitude of any potential bias<br>Done: "The most important limitation of our study is the use of register-based approximations of RIA. While our definitions of *low social class*, *large family size*, *paternal criminality*, *maternal mental disorder*, and *placement in out-of-home care* are quite similar to those used in other studies, the definition of *severe marital discord* differs more substantially. Since the registers do not contain information about the degree of conflicts among cohabiting individuals, we operationalized this particular variable dichotomously according to whether both custodial parents were living at the same address as the infant or not. Based on the present data, we are unable to determine whether this definition captures the same construct as that originally defined by Rutter and colleagues [10-12]. However, this is little different from other studies of RIA and ADHD [13-16], which reveal that there is currently no consensus regarding the definition of *severe marital discord*.
Interpretation	20	Give a cautious overall interpretation of results considering objectives, limitations, multiplicity of analyses, results from similar studies, and other relevant evidence<br>Done: See results section.
Generalisability	21	Discuss the generalisability (external validity) of the study results<br>Done: "In terms of generalizability, it is important to note that Denmark is among the most economically and socially equal welfare states in the world [41], and the strong association between RIA assessed in infancy and ADHD documented in this study may therefore not be representative for societies providing other levels of welfare to its citizens. However, if RIA-ADHD associations of similar strength exist in less developed societies, the ADHD-predictive potential of the RIA will be even more pronounced from a public health perspective, under the assumption that a relatively larger proportion of children will be growing up under psychosocially adverse circumstances (high RIA-scores) in such societies."
**Other information**		
Funding	22	Give the source of funding and the role of the funders for the present study and, if applicable, for the original study on which the present article is based<br>Done: "The study was supported by grants from the Lundbeck Foundation. The funders of the study had no role in study design, data collection, data analysis, data interpretation, patient recruitment, writing of the paper, or the decision to submit for publication."

*Give information separately for cases and controls in case-control studies and, if applicable, for exposed and unexposed groups in cohort and cross-sectional studies.

Note: An Explanation and Elaboration article discusses each checklist item and gives methodological background and published examples of transparent reporting. The STROBE checklist is best used in conjunction with this article (freely available on the Web sites of PLoS Medicine at http://www.plosmedicine.org/, Annals of Internal Medicine at http://www.annals.org/, and Epidemiology at http://www.epidem.com/). Information on the STROBE Initiative is available at www.strobe-statement.org.
