# Supplementary material for: Predicting ADHD by Assessment of Rutter’s Indicators of Adversity in Infancy
Source: PLoS One. 2016 Jun 29;11(6):e0157352. doi: 10.1371/journal.pone.0157352 (PMC4927115; doi:10.1371/journal.pone.0157352)
Supplement: S1 Table — ”N ASD” refers to the number of ASD cases in each stratum, while”N total” is the total number of individuals in each stratum. The rates are per 1,000 person-years. The hazard ratios (HR) are adjusted for calendar year (1 year strata). Information regarding low social class, severe marital discord and large family size was unavailable for 0.03%, 0.16%, and 0.16% of the ASD cases respectively. For the entire cohort, the corresponding proportions were 0.07%, 0.21% and 0.21%. (DOCX) [file pone.0157352.s002.docx]

**S1 Table.** Incidence rates and adjusted hazard ratios for ASD for each of Rutter’s indicators of adversity (RIA) and for the total RIA-score assessed in infancy

|  |  | **N autism** | **(%)** | **N total** | **(%)** | **Rate (95% CI)** | **HR (95% CI)** |
| --- | --- | --- | --- | --- | --- | --- | --- |
| **FEMALES** |  |  |  |  |  |  |  |
| **Low social class** | No | 2,443 | (86.02) | 440,316 | (90.82) | 0.52 (0.50-0.55) | 1.00 (ref) |
|  | Yes | 394 | (13.87) | 44,136 | (9.10) | 0,75 (0.68-0.83) | 1.42 (1.28-1.58) |
| **Severe marital discord** | No | 2,411 | (84.89) | 435,848 | (89.90) | 0.52 (0.50-0.54) | 1.00 (ref) |
|  | Yes | 424 | (14.93) | 47,924 | (9.89) | 0.78 (0.71-0.85) | 1.49 (1.34-1.65) |
| **Large family size** | No | 2,751 | (96.87) | 466,615 | (96.25) | 0.55 (0.53-0.57) | 1.00 (ref) |
|  | Yes | 84 | (2.96) | 17,157 | (3.54) | 0.46 (0.37-0.57) | 0.83 (0.67-1.03) |
| **Paternal criminality** | No | 2,480 | (87.32) | 433,081 | (89.33) | 0.54 (0.51-0.56) | 1.00 (ref) |
|  | Yes | 360 | (12.68) | 51,718 | (10.67) | 0.65 (0.59-0.72) | 1.21 (1.08-1.35) |
| **Maternal mental disorder** | No | 2,633 | (92.71) | 458,291 | (94.53) | 0.53 (0.51-0.55) | 1.00 (ref) |
|  | Yes | 207 | (7.29) | 26,508 | (5.47) | 0.98 (0.86-1.13) | 1.92 (1.67-2.22) |
| **Out-of-home care** | No | 2,811 | (98.98) | 483,532 | (99.74) | 0.54 (0.52-0.56) | 1.00 (ref) |
|  | Yes | 29 | (1.02) | 1,267 | (0.26) | 2.23 (1.55-3.22) | 4.15 (2.88-5.98) |
| **RIA-score** | 0 | 1,822 | (64.15) | 348,965 | (71.98) | 0.49 (0.47-0.51) | 1.00 (ref) |
|  | 1 | 669 | (23.56) | 96,304 | (19.86) | 0.64 (0.60-0.70) | 1.32 (1.21-1.44) |
|  | 2 | 244 | (8.59) | 28,445 | (5.87) | 0.78 (0.69-0.89) | 1.60 (1.40-1.83) |
|  | 3 | 83 | (2.92) | 9,037 | (1.86) | 0.85 (0.69-1.06) | 1.75 (1.40-2.17) |
|  | 4 | 18 | (0.63) | 1,836 | (0.38) | 1.00 (0.63-1.59) | 2.06 (1.29-3.28) |
|  | 5-6 | 4 | (0.14) | 212 | (0.04) | 1.91 (0.72-5.08) | 3.92 (1.47-10.46) |
| **MALES** |  |  |  |  |  |  |  |
| **Low social class** | No | 8,643 | (86.13) | 463,755 | (90.78) | 1.77 (1.73-1.81) | 1.00 (ref) |
|  | Yes | 1,391 | (13.86) | 46,714 | (9.14) | 2.52 (2.39-2.65) | 1.48 (1.39-1.56) |
| **Severe marital discord** | No | 8,568 | (85.38) | 458,995 | (89.85) | 1.77 (1.73-1.80) | 1.00 (ref) |
|  | Yes | 1,451 | (14.46) | 50,832 | (9.95) | 2.53 (2.40-2.66) | 1.46 (1.38-1.54) |
| **Large family size** | No | 9,703 | (96.69) | 491,721 | (96.26) | 1.85 (1.82-1.89) | 1.00 (ref) |
|  | Yes | 316 | (3.15) | 18,106 | (3.54) | 1.64 (1.47-1.83) | 0.88 (0.79-0.98) |
| **Paternal criminality** | No | 8,687 | (86.57) | 456,620 | (89.38) | 1.79 (1.75-1.83) | 1.00 (ref) |
|  | Yes | 1,348 | (13.43) | 54,230 | (10.62) | 2.34 (2.22-2.47) | 1.31 (1.24-1.39) |
| **Maternal mental disorder** | No | 9,263 | (92.31) | 483,143 | (94.58) | 1.78 (1.74-1.81) | 1.00 (ref) |
|  | Yes | 772 | (7.69) | 27,707 | (5.42) | 3.55 (3.31-3.81) | 1.93 (1.79-2.08) |
| **Out-of-home care** | No | 9,951 | (99.16) | 509,581 | (99.75) | 1.83 (1.80-1.87) | 1.00 (ref) |
|  | Yes | 84 | (0.84) | 1,269 | (0.25) | 6.44 (5.20-7.97) | 3.53 (2.85-4.38) |
| **RIA-score** | 0 | 6,379 | (63.57) | 367,454 | (71.93) | 1.64 (1.60-1.68) | 1.00 (ref) |
|  | 1 | 2,426 | (24.18) | 101,968 | (19.96) | 2.22 (2.13-2.31) | 1.37 (1.31-1.43) |
|  | 2 | 857 | (8.54) | 29,763 | (5.83) | 2.66 (2.49-2.85) | 1.65 (1.54-1.78) |
|  | 3 | 289 | (2.88) | 9,529 | (1.87) | 2.84 (2.53-3.19) | 1.76 (1.57-1.98) |
|  | 4 | 65 | (0.65) | 1,906 | (0.37) | 3.59 (2.82-4.58) | 2.17 (1.70-2.77) |
|  | 5-6 | 19 | (0.19) | 230 | (0.05) | 8.72 (5.56-13.66) | 5.25 (3.35-8.24) |

”N ASD” refers to the number of ASD cases in each stratum, while ”N total” is the total number of individuals in each stratum. The rates are per 1,000 person-years. The hazard ratios (HR) are adjusted for calendar year (1 year strata). Information regarding low social class, severe marital discord and large family size was unavailable for 0.03%, 0.16%, and 0.16% of the ASD cases respectively. For the entire cohort, the corresponding proportions were 0.07%, 0.21% and 0.21%.
