# Supplementary material for: Predicting ADHD by Assessment of Rutter’s Indicators of Adversity in Infancy
Source: PLoS One. 2016 Jun 29;11(6):e0157352. doi: 10.1371/journal.pone.0157352 (PMC4927115; doi:10.1371/journal.pone.0157352)
Supplement: S2 Table — The number needed to screen was calculated as one divided by the difference between the risk of ADHD among cohort members with an increased RIA-score and those with a RIA-score equal to zero [30]. The risk of ASD was estimated as one minus the Kaplan-Meier estimator. (DOCX) [file pone.0157352.s003.docx]

**S2 Table.** The number needed to screen to detect one case of ASD based on the Rutter’s Indicators of adversity score (RIA-score) assessed in infancy

|  |  | **Number needed to screen (95% CI)** | | | |
| --- | --- | --- | --- | --- | --- |
|  |  | **Prior to age 5** | **Prior to age 10** | **Prior to age 15** | **Prior to age 20** |
| **FEMALES** |  |  |  |  |  |
| **RIA-score** | **1** | 3,933.55 (2,558.50-6,683.44) | 996.89 (788.52-1,289.94) | 512.82 (415.08-648.18) | 251.63 (206.98-311.16) |
|  | **2** | 2,062.19 (1,106.94-4,933.10) | 490.21 (350.58-720.62) | 212.07 (162.64-285.54) | 158.92 (117.54-225.53) |
|  | **3** | 1,255.09 (547.66-4,568.17) | 379.27 (222.12-757.72) | 187.24 (118.20-337.75) | 116.87 (73.06-214.15) |
|  | **4** | 339.92 (133.80-1,055.37) | 202.42 (89.60-628.93) | 104.81 (49.10-317.33) | 130.38 (51.25-1,451.25) |
|  | **5-6** | 73.31 (23.09-253.82) | 54.50 (18.76-192.51) | 71.87 (20.55-1,058.24) | 101.49 (22.53-344.00) |
|  |  |  |  |  |  |
| **MALES** |  |  |  |  |  |
| **RIA-score** | **1** | 776.69 (647.72-942.61) | 200.66 (180.89-223.54) | 125.69 (114.55-138.41) | 85.24 (77.67-93.85) |
|  | **2** | 419.71 (317.14-574.19) | 138.86 (114.60-171.57) | 65.96 (57.17-76.82) | 48.30 (41.90-56.17) |
|  | **3** | 375.22 (230.84-699.54) | 105.51 (78.10-150.38) | 53.27 (41.94-69.76) | 44.11 (34.50-58.28) |
|  | **4** | 175.42 (87.62-432.79) | 58.32 (36.01-106.86) | 30.25 (20.11-49.33) | 26.29 (16.79-45.84) |
|  | **5-6** | 48.43 (18.78-145.22) | 16.13 (8.75-32.52) | 10.99 (6.31-21.00) | 7.14 (3.95-14.45) |

The number needed to screen was calculated as one divided by the difference between the risk of ADHD among cohort members with an increased RIA-score and those with a RIA-score equal to zero [35]. The risk of ASD was estimated as one minus the Kaplan-Meier estimator.
