# Supplementary material for: Predicting ADHD by Assessment of Rutter’s Indicators of Adversity in Infancy
Source: PLoS One. 2016 Jun 29;11(6):e0157352. doi: 10.1371/journal.pone.0157352 (PMC4927115; doi:10.1371/journal.pone.0157352)
Supplement: S3 Table — The number needed to screen was calculated as one divided by the difference between the risk of ASD among cohort members with an increased RIA-score and those with a RIA-score equal to zero [30]. The risk of ASD was estimated as one minus the Kaplan-Meier estimator. In this analysis (compared to that providing the results described in S2 Table), cohort members registered with a diagnosis of ADHD prior to initiation of follow-up for ASD were excluded. Furthermore, receiving a diagnosis of ADHD was included among the events censoring follow-up for ASD. (DOCX) [file pone.0157352.s004.docx]

**S3 Table.** ADHD-adjusted number needed to screen to detect one case of ASD based on the Rutter’s Indicators of adversity score (RIA-score) assessed in infancy

|  | | **Number needed to screen (95% CI)** | | | |
| --- | --- | --- | --- | --- | --- |
|  |  | **Prior to age 5** | **Prior to age 10** | **Prior to age 15** | **Prior to age 20** |
| **FEMALES** |  |  |  |  |  |
| **RIA-score** | **1** | 4,399.63 (2,773.90-7,928.55) | 1,167.57 (902.89-1,556.77) | 670.72 (521.80-895.18) | 280.51 (226.15-355.70) |
|  | **2** | 2,147.09 (1,137.26-5,303.23) | 526.58 (371.55-788.40) | 227.46 (172.65-310.19) | 155.10 (114.68-219.53) |
|  | **3** | 1,421.23 (585.12-6,564.45) | 468.89 (256.26-1,090.85) | 243.44 (142.31-516.79) | 237.34 (122.64-766.14) |
|  | **4** | 425.82 (149.30-1,711.59) | 217.01 (92.40-732.70) | 153.58 (62.36-845.55) | 193.88 (62.01-1,057.11) |
|  | **5-6** | 112.36 (26.72-587.29) | 71.14 (20.30-359.23) | 99.82 (22.20-926.54) | 157.27 (24.28-219.96) |
|  |  |  |  |  |  |
| **MALES** |  |  |  |  |  |
| **RIA-score** | **1** | 810.88 (672.83-990.13) | 218.89 (196.23-245.33) | 144.91 (130.89-161.17) | 102.00 (91.80-113.86) |
|  | **2** | 420.45 (317.78-574.81) | 148.40 (121.54-185.09) | 77.82 (66.30-92.49) | 55.79 (47.71-65.97) |
|  | **3** | 437.25 (256.30-903.81) | 123.66 (88.48-185.68) | 72.25 (53.75-102.61) | 55.56 (41.26-78.98) |
|  | **4** | 170.87 (86.00-412.70) | 57.89 (35.50-106.45) | 28.76 (19.05-46.77) | 25.07 (15.79-44.20) |
|  | **5-6** | 46.26 (18.02-136.71) | 17.38 (8.86-38.16) | 14.31 (7.42-31.93) | 9.32 (4.20-25.96) |

The number needed to screen was calculated as one divided by the difference between the risk of ASD among cohort members with an increased RIA-score and those with a RIA-score equal to zero [35]. The risk of ASD was estimated as one minus the Kaplan-Meier estimator. In this analysis (compared to that providing the results described in S2 Table), cohort members registered with a diagnosis of ADHD prior to initiation of follow-up for ASD were excluded. Furthermore, receiving a diagnosis of ADHD was included among the events censoring follow-up for ASD.
