# Supplementary material for: Predicting ADHD by Assessment of Rutter’s Indicators of Adversity in Infancy
Source: PLoS One. 2016 Jun 29;11(6):e0157352. doi: 10.1371/journal.pone.0157352 (PMC4927115; doi:10.1371/journal.pone.0157352)
Supplement: S1 Text — (DOCX) [file pone.0157352.s005.docx]

**Supporting Text**

**Supplemental Methods**

For the analysis of the association between RIA and ASD, the same birth cohort and the same analytic approach (Cox regression and calculation of number needed to screen (NNS))) was used as for the analysis of the association between RIA and ADHD, with the following modifications: Individuals receiving an ICD-10 diagnosis of F84.0, F84.1, F84.5, F84.8, or F84.9 following either inpatient or outpatient treatment were defined as ASD cases. Since ASD is more likely to have its onset prior to the age of 2 (which represented starting point for follow-up in the analysis of ADHD), we began follow-up on the cohort members’ first birthday (or on January 1^st^ 1995 for those born in 1993) and ended it at the occurrence of the first of the following events: a diagnosis of ASD, emigration, death or December 31^st^ 2013. Individuals registered with a diagnosis of ASD (defined by an ICD-10 diagnosis of F84.0, F84.1, F84.5, F84.8, or F84.9 - or an ICD-8 diagnosis of 299.00, 299.01, 299.02, or 299.03) before the beginning of follow-up were excluded (n=46).

**Supplemental Results**

We identified 1,000,296 children born between January 1^st^ 1993 and December 31^st^ 2011 to Danish born parents. Of these, 4,740 either died (n=4,170), emigrated / were lost to follow-up (n=524), or received an ASD diagnosis (n=46) before their first birthday or January 1^st^ 1995. Thus, 995,649 children (510,850 males and 484,799 females) were followed from their 1-year birthday or from January 1^st^ 1995, yielding a total of 10,624,755 person-years of observation. During follow-up, 10,035 males and 2,840 females from the cohort were diagnosed with ASD, corresponding to incidence rates of 1.85 (95%CI: 1.81-1.88) and 0.55 (95%CI: 0.53-0.57) per 1,000 person-years for males and females respectively. S1 Table shows incidence rates and hazard ratios for the development of ASD for each of the six RIA, as well as for the summed RIA, stratified on males and females.
